# Supplementary material for: Genetic and genomic analyses of Drosophila melanogaster models of chromatin modification disorders
Source: Genetics. 2023 Apr 10;224(4):iyad061. doi: 10.1093/genetics/iyad061 (PMC10411607; doi:10.1093/genetics/iyad061)
Supplement: iyad061_Supplementary_Data [file iyad061_supplementary_data.zip › Figure_S1_GENETICS-2023-306034.pdf]

Fly Gene Symbol	BDRC Line #	*GAL4* Driver Line: Ubiquitin	*GAL4* Driver Line: Actin	*GAL4* Driver Line: Ubi156
Bap111	35242	embryo	adult	✕
brm	34520	✕	✕	✕
brm	35211	adult	adult	adult
Nipped-B	32406	larva	adult	pupa
osa	35447	larva	adult	adult
SMC3	60017	✕	adult	adult
SMC3	33431	embryo	✕	✕
SMC1	34351	adult	adult	adult
SMC1	36598	adult	adult	adult
Snr1	32372	larva	✕	adult
vtd	36786	larva	adult	adult

embryo

larva

pupa

adult

✕ escaper flies and/or no knockdown
