# Supplementary material for: Genetic and genomic analyses of Drosophila melanogaster models of chromatin modification disorders
Source: Genetics. 2023 Apr 10;224(4):iyad061. doi: 10.1093/genetics/iyad061 (PMC10411607; doi:10.1093/genetics/iyad061)
Supplement: iyad061_Supplementary_Data [file iyad061_supplementary_data.zip › Supplemental_Material_Legends_GENETICS-2023-306034.docx]

**Supplementary Information**

**Figure S1. Gross viability observations in potential CSS/NCBRS and CdLS fly models.** Life stage shown is the final stage of the *Drosophila* life cycle where live individuals were observed. “X” indicates flies did not have detectable levels of gene knockdown, as quantified via qRT-PCR.

**Figure S2. Selection of genes for k-means clustering.** Elbow plots of maximal fold change in expression plotted against rank order (blue) across all analyses for each of 9657 genes (A) and for genes with a maximum fold change difference greater than 4 (B). See Table S7. The red and green lines were fit to roughly linear segments of the generated distribution (blue). The orange lines are drawn from the plot elbow (determined by the x coordinate of the intersection of the green and red lines) to the x and y axes.

**Figure S3. Overlap of differentially expressed genes in SSRIDD and CdLS fly models.** Venn diagrams displaying the number of differentially expressed genes (FDR < 0.05), in **SSRIDD** and CdLS fly models, sexes separately. Pairwise gene-specific analyses from (A) CdLS fly models and (B) **SSRIDD** fly models. Panel (C) shows overlap of disease-specific analyses, pooled across disease-associated genes.

**Figure S4. Altered phenotypes due to knockdown of co-regulated genes.** Bar plots displaying differences in the average values of the experimental line versus the control line for (A) startle response, (B) percent of flies tapping, (C) total activity, and (D) proportion of time asleep at night. All lines have *Ubi156-GAL4*-mediated RNAi knockdown. Females and males are shown in purple and green, respectively. See Table S14 for ANOVAs (A,B,D) and Fishers Exact Tests (C). N=29-32 per sex per line. Error bars represent standard error of the difference based on error propagation (Burns and Dobson 1981). Asterisks represent pairwise analyses of the experimental line vs the control, sexes separately. *: *p* < 0.05, **: *p* < 0.01, ***: *p* < 0.001.

**Table S1. Fly reagents and primer sequences.** Drosophila reagents and primer sequences. (A) Drosophila lines used. (B) Primer sequences used for qRT-PCR. BDSC: Bloomington Drosophila Stock Center.

**Table S2. Ortholog prediction scores for potential focal genes.** Human-Drosophila ortholog prediction scores generated using Drosophila RNAi Screening Center Integrative Ortholog Prediction Tool (DIOPT). Human genes associated with SSRIDDs and Cornelia de Lange syndrome.

**Table S3. Percent knockdown of focal genes.** Average RNAi-mediated qRT-PCR knockdown of focal genes.

**Table S4. Quantification of changes in behavior and brain morphology from knockdown of focal genes.** Quantification of changes in behavior and brain morphology from RNAi knockdown. Statistical analyses characterizing SSRIDD and CdLS fly models. (A) ANOVAs for startle response. (B) Fisher's Exact Tests for tapping behavior. (C) ANOVAs for sleep and activity measurements. (D) ANOVAS for mushroom body lobe lengths. (E) Levene's and Brown-Forsythe Tests for unequal variances of mushroom body lobe length data. (F) Gross brain abnormalities. Line and Sex are fixed effects. df: degrees of freedom, SS: Type III Sum of Squares, MS: Mean Squares.

**Table S5. ANOVA results from differential expression analyses.** Gene name, gene symbol, FlyBase ID, normalized read counts (counts per million), and raw and Benjamini-Hochberg FDR adjusted *p*-values for all genes for all model terms used in the ANOVA analyses. (A) Full model using all knockdown lines and the control according to the model *Y = μ + Line + Sex + Line x Sex + ɛ* for 15915 genes. (B-G) Pairwise comparisons of single gene knockdown vs. the control (sexes together *Y = μ + Line + Sex + Line x Sex + ɛ*; and sexes separately *Y = μ + Line + ɛ*) on the 9657 genes from the full model differentially expressed (FDR < 0.05) for the *Line* and/or *Line x Sex* terms. (B) *brm*. (C) *osa*. (D) *Snr1*. (E) *SMC1*. (F) *SMC3*. (G) *vtd*. (H-I) Disease-specific comparisons (sexes together *Y = μ + Line + Sex + Line x Sex + ɛ*; and sexes separately *Y = μ + Line + ɛ*). (H) SSRIDDs. (I) Cornelia de Lange syndrome (CdLS).

**Table S6. Overlap of differentially expressed genes across analyses.** FDR-corrected *p*-values less than 0.05 for the *Line* term of each of the 9657 genes. (A) Pairwise analyses of each knockdown line compared to the control, sexes separately. (B) Disease-specific analyses, sexes separately. NA indicates FDR-corrected *P*-values for the effect of *Line* greater than 0.05.

**Table S7. k-means threshold.** (A) Average log2 fold change values for each differentially expressed gene for each set of samples, as well as maximum, minimum across all samples. (B) Determination of threshold by ranking, indexing and fitting lines to fold change plots. fc: log2 fold change; f: females, m: males.

**Table S8. k-means clustering gene lists.** Lists of genes within each k-means cluster. (A) Females. (B) Males.

**Table S9. Gene Ontology (GO) analyses for differentially expressed genes.** “Analysis” indicates the gene set used in the analysis.

**Table S10. Gene Ontology (GO) analyses for k-means clusters.** “Analysis” indicates the gene set used in the analysis.

**Table S11. Ortholog prediction scores for differentially expressed genes**. Drosophila-human ortholog prediction scores, generated using Drosophila RNAi Screening Center Integrative Ortholog Prediction Tool (DIOPT). Differentially expressed fly genes for each by-sex pairwise comparison.

**Table S12. Ortholog prediction scores and known disease associations for co-regulated genes.** Drosophila-human ortholog prediction scores, generated using Drosophila RNAi Screening Center Integrative Ortholog Prediction Tool (DIOPT) and Online Mendelian Inheritance of Man (OMIM)-derived known disease/phenotype associations and corresponding MIM numbers. Subset of 31 Drosophila genes co-regulated with *brm*, *osa*, and/or *Snr1*.

**Table S13. Percent knockdown of co-regulated genes.** Average RNAi-mediated qRT-PCR knockdown of co-regulated genes.

**Table S14. Quantification of changes in behavior from knockdown of co-regulated genes.** Quantification of changes in behavior from RNAi knockdown of co-regulated genes. (A) ANOVAs for startle response. (B) Fisher's Exact Tests for tapping behavior. (C) ANOVAs for sleep and activity measurements. Line and Sex are fixed effects. df: degrees of freedom, SS: Type III Sum of Squares, MS: Mean Squares.

**File S1. Video of tapping behavior in a male fly with knockdown of *vtd* following a startle response.**

**File S2. Video of control male fly following a startle response.**
