# Supplementary figures and images for: Genetic and genomic analyses of Drosophila melanogaster models of chromatin modification disorders
Source: Genetics. 2023 Apr 10;224(4):iyad061. doi: 10.1093/genetics/iyad061 (PMC10411607; doi:10.1093/genetics/iyad061)

A

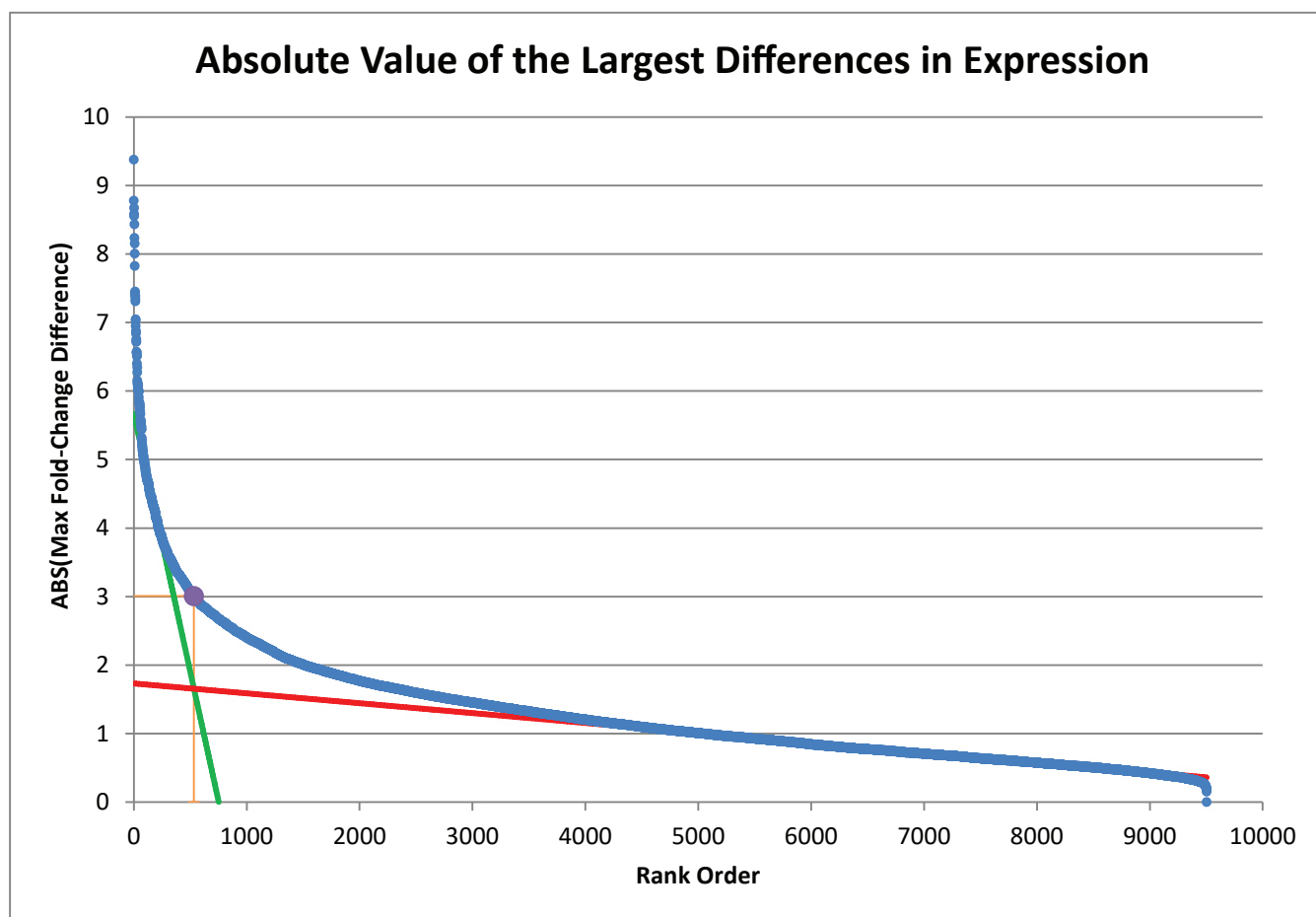

B

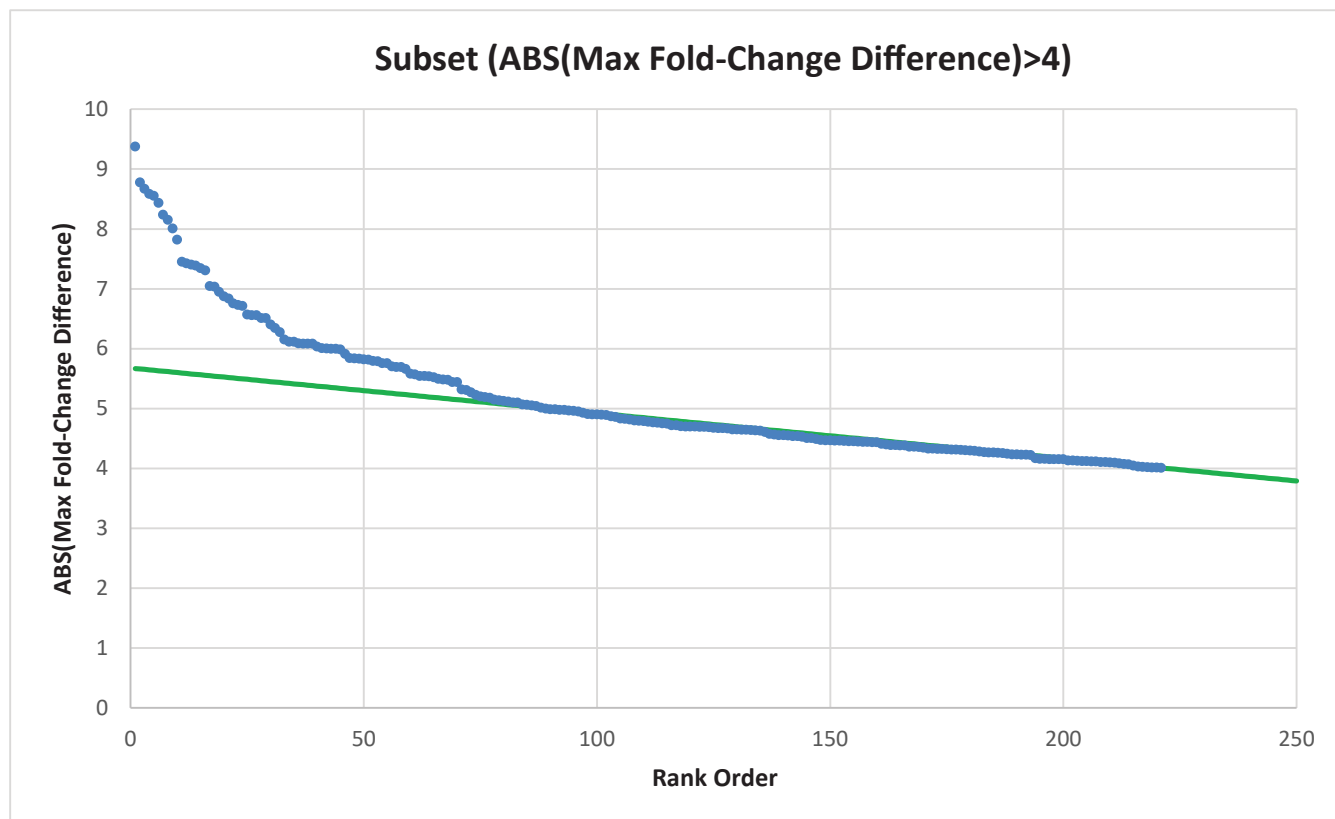

Supplement: iyad061_Supplementary_Data [file iyad061_supplementary_data.zip › Figure_S2_GENETICS-2023-306034.pdf]

**A.**

CdLS Females

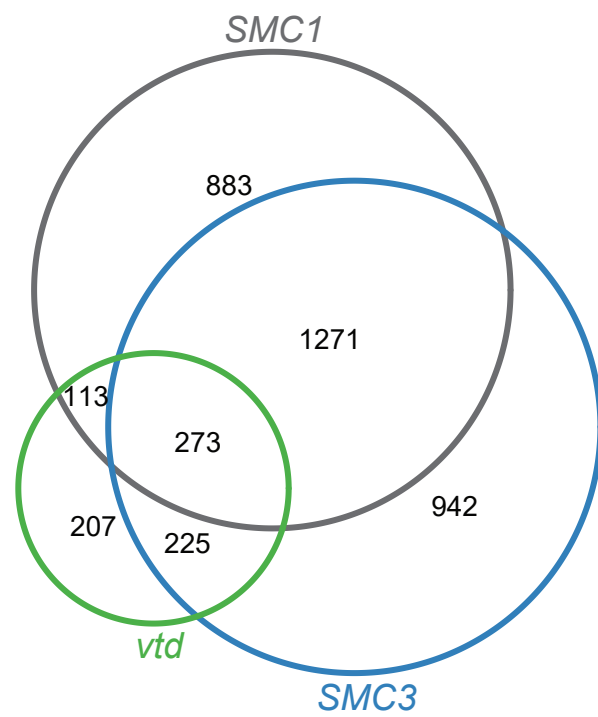

CdLS Males

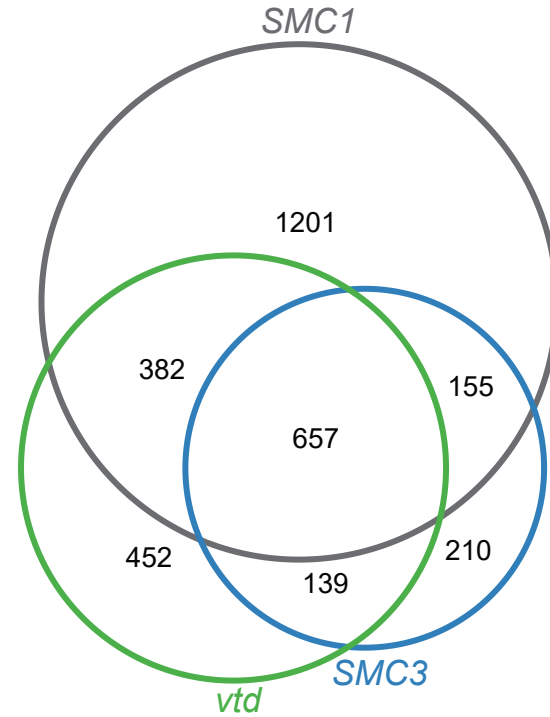**B.**

SSRIDD Females

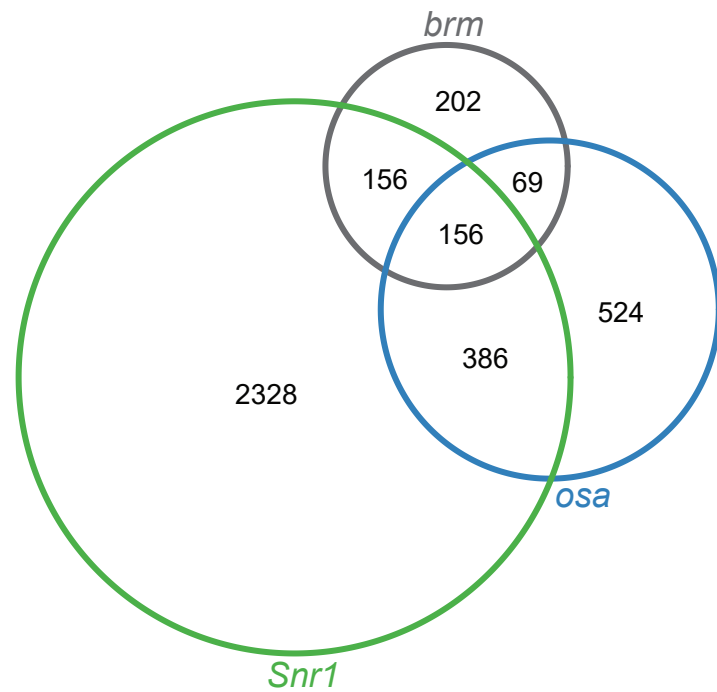

SSRIDD Males

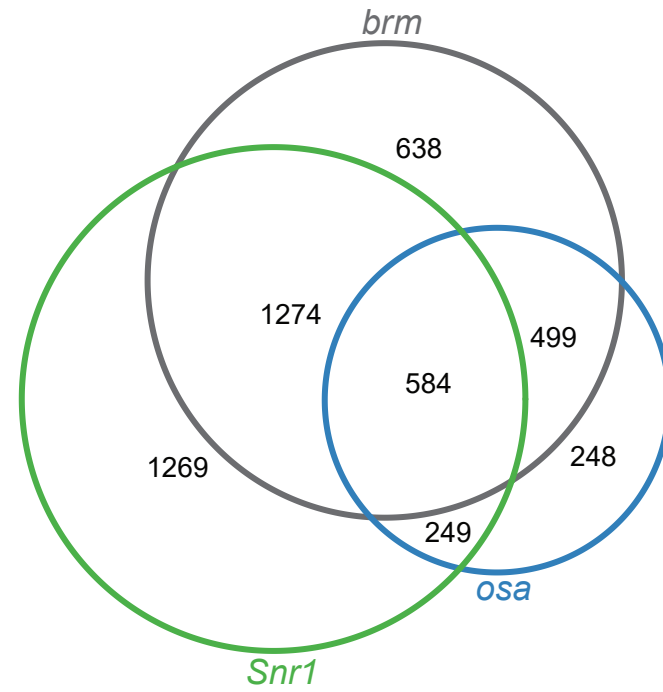**C.**

SSRIDD vs CdLS

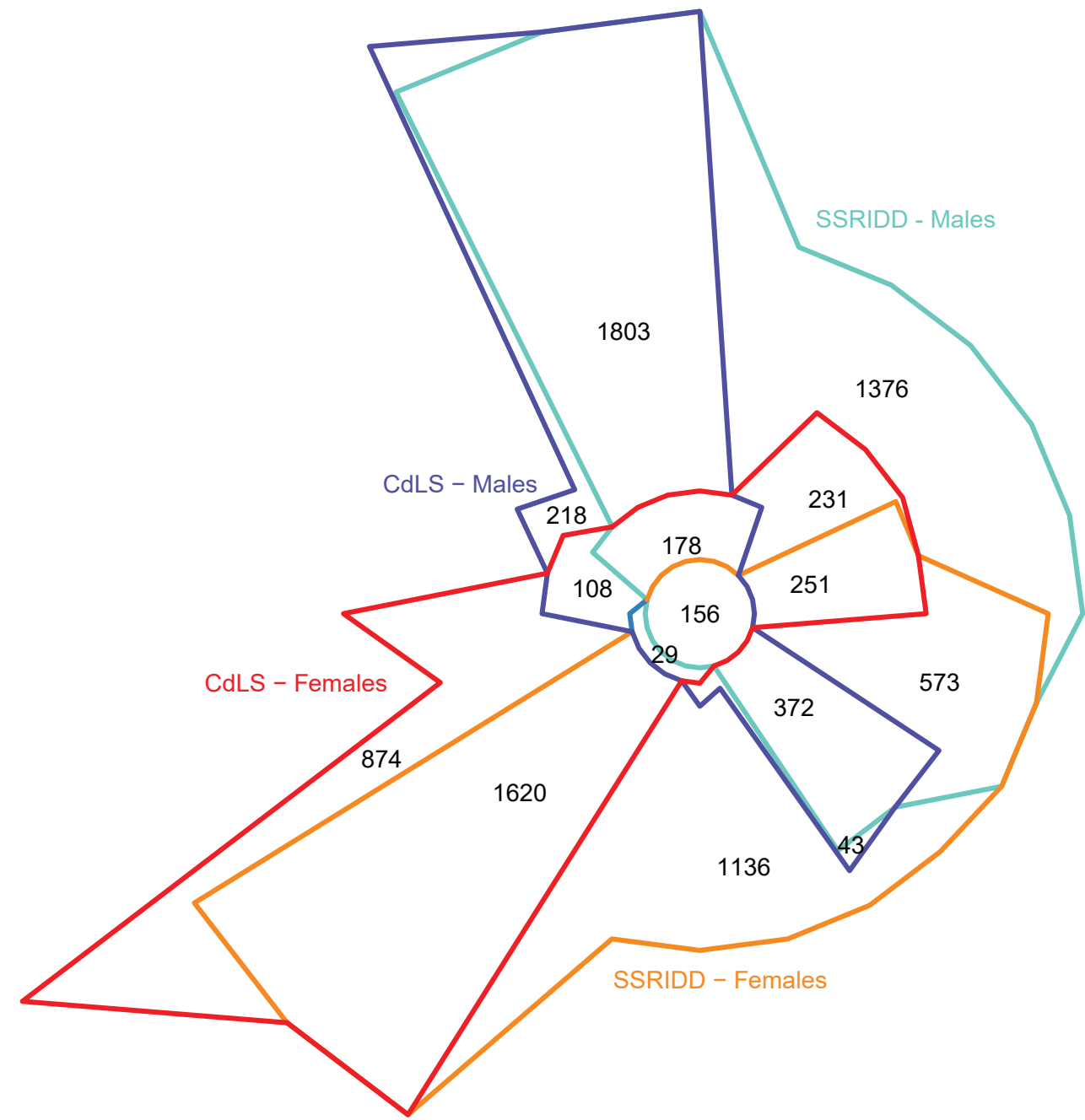

Supplement: iyad061_Supplementary_Data [file iyad061_supplementary_data.zip › Figure_S3_GENETICS-2023-306034.pdf]

**A**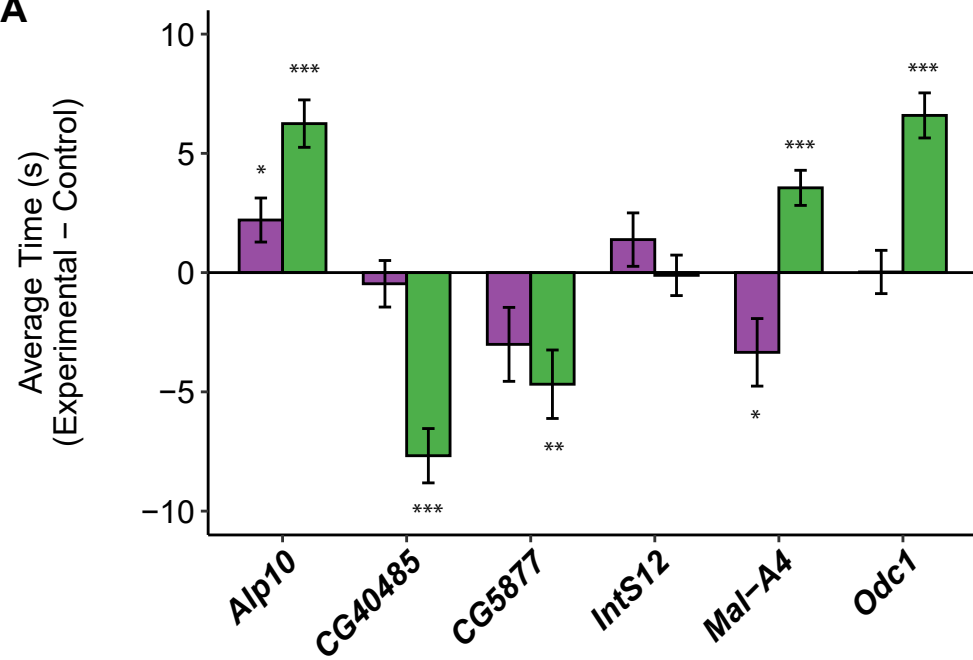**B**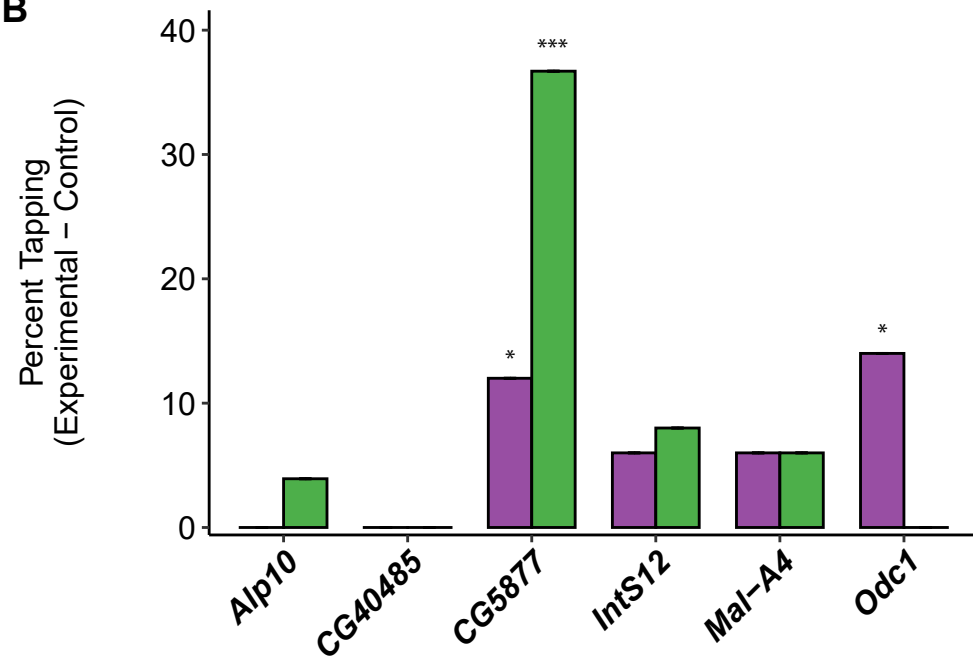**C**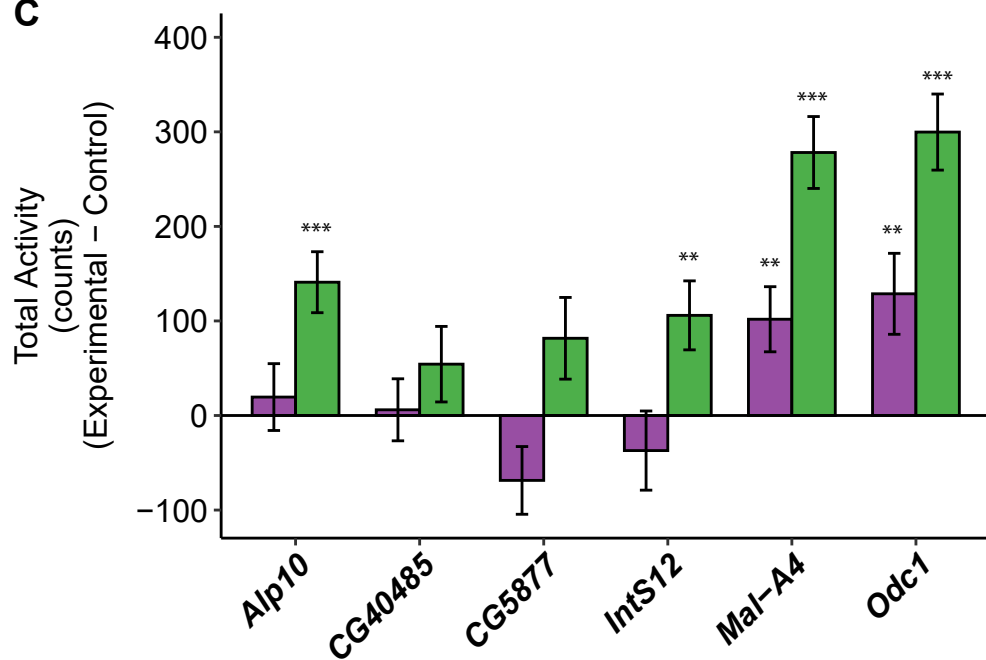**D**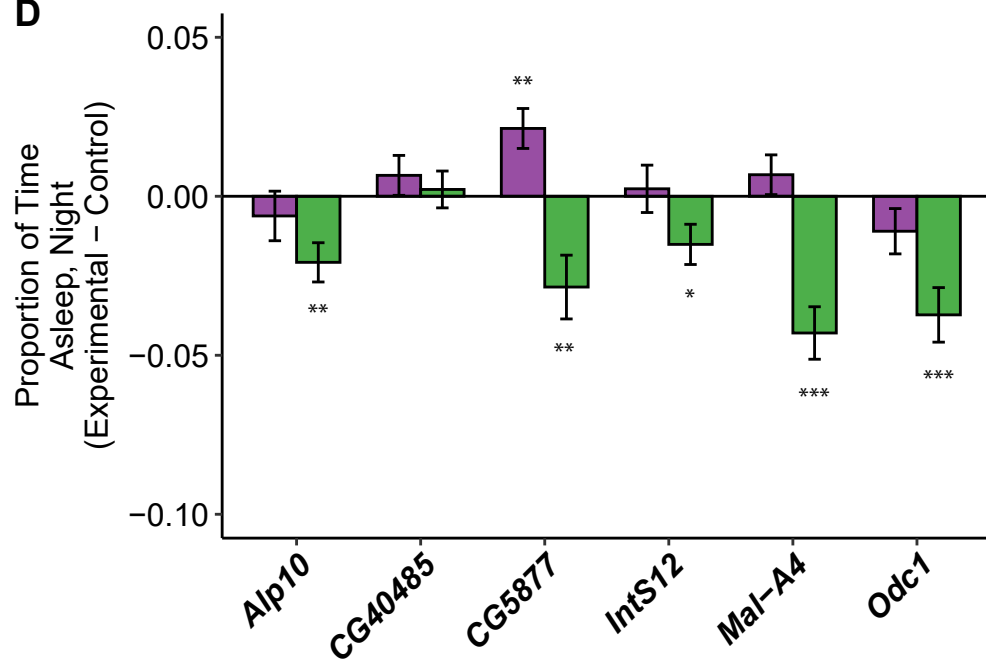

Supplement: iyad061_Supplementary_Data [file iyad061_supplementary_data.zip › Figure_S4_GENETICS-2023-306034.pdf]
